# ERBB3 (HER3) is a key sensor in the regulation of ERBB-mediated signaling in both low and high ERBB2 (HER2) expressing cancer cells

**DOI:** 10.1002/cam4.10

**Published:** 2012-07-15

**Authors:** Byung-Kwon Choi, Xuejun Fan, Hui Deng, Ningyan Zhang, Zhiqiang An

**Affiliations:** Texas Therapeutics Institute, Brown Foundation Institute of Molecular Medicine, University of Texas Health Science Center at HoustonHouston, Texas

**Keywords:** Anti-HER2 antibody, EGFR, ERBB2/HER2, ERBB3/HER3, MCF7, signaling

## Abstract

Aberrant expression and activation of EGFR and ERBB2 (HER2) have been successfully targeted for cancer therapeutics. Recent evidence from both basic and clinical studies suggests that ERBB3 (HER3) serves as a key activator of downstream signaling through dimerization with other ERBB proteins and plays a critical role in the widespread clinical resistance to EGFR and HER2 targeting cancer therapies. As a result, HER3 is actively pursued as an antibody therapeutic target for cancer. Ligand binding is thought to be a prerequisite for dimerization of HER3 with other ERBB proteins, which results in phosphorylation of its c-terminal tyrosine residues and activation of downstream AKT and MAPK signaling pathways. In this study, we report that an anti-HER2 monoclonal antibody (HER2Mab), which blocks HER2 dimerization with HER3, induces HER3 dimerization with EGFR in both low and high HER2 expressing cancer cells. Treatment of the low HER2 expressing MCF7 cancer cells with HER2Mab promoted cell proliferation and migration in the absence of HER3 ligand stimulation. Follow-up studies revealed that HER2Mab-induced HER3 signaling via EGFR/HER3 dimerization and activation of downstream AKT signaling pathways. These results suggest that equilibrium of dimerization among the ERBB proteins can be perturbed by HER2Mab and HER3 plays a key role in sensing the perturbation.

## Introduction

The epidermal growth factor receptor (EGFR/HER/ERBB) family contains four closely related type 1 transmembrane receptor tyrosine kinases (RTK) [1, 2]. EGFR, ERBB3/HER3, and ERBB4/HER4 exist in tethered (close) conformation as inactive forms and typically require ligand binding for conformational change to induce dimerization and downstream signaling, whereas ERBB2/HER2 has no reported ligands and exists in a constitutively open conformation for dimerization and activation [2]. Both EGFR and HER2 are well studied, but our understanding on the regulatory function of HER3 signaling remains limited. Emerging studies suggest that HER3 which lacks kinase activity is a preferred partner for dimerization among the receptors and HER2/HER3 partnership constitutes the most oncogenic unit [3]. Ligand-independent HER2/HER3 dimerization was reported recently [4]. However, it is not clear whether the ligand-independent HER dimerization only occurs in an oncogenic high ERBB expression context such as cancer cells with high HER2 expression, or it can occur in low HER2 expressing cell lines.

Aberrant expression and activation of the ERBB proteins are oncogenic. Both EGFR and HER2 have been successfully targeted for cancer therapeutics including small molecule kinase inhibitors (such as gefitinib, erlotinib, and lapatinib) and therapeutic monoclonal antibodies (such as cetuximab, panitumumab, and trastuzumab) [2, 3, 5]. However, drug resistance to the ERBB targeted therapies is widespread due to both innate gene mutations and compensatory signaling among the ERBB family of receptors [6]. Humanized anti-HER2 antibody rhuMAB 2C4 or pertuzumab (IMGT/mAb-DB, http://www.imgt.org) is an monoclonal antibody that blocks HER2 dimerization with other ERBB family members in HER2 overexpressing cancer cells [7], and the antibody has been reported to have no efficacy in cancer cells expressing low levels of HER2, such as MCF7 breast cancer cells [8, 9]. To understand the role of HER3 plays in response to anti-HER2 antibody treatment in both low and high HER2 expression cancer cells, we constructed a monoclonal antibody named HER2Mab using the variable domain sequences of rhuMAB 2C4 and the antibody was expressed in HEK293 cells. The results reported here show that HER2Mab significantly promoted proliferation and migration of the low HER2 expressing MCF7 breast cancer cells. Using an in situ receptor dimerization detection method known as the proximity ligation assay (PLA), we showed that HER2Mab promoted ligand-independent HER3 signaling via EGFR/HER3 dimerization and activation of downstream AKT signaling pathways.

## Materials and Methods

### Cell lines, antibodies, and other reagents

RPMI 1640 media, l-glutamine were purchased from Life Technologies (Carlsbad, CA). Fetal bovine serum (FBS) was obtained from Hyclone (Logan, UT). Neuregulin 1 (NRG1) was from R&D systems (Minneapolis, MN). HER3/ERBB3 Phospho (pY1289) RabMab, EGFR (C-term) RabMab, AKT1 RabMab, AKT1 Phospho (pS473) RabMab, p44 MAPK (ERK1) (N-term) RabMab, and ERK1 Phospho (pY204)/ERK2 Phospho (pY187) RabMab were from Epitomics (Burlingame, CA). Antibodies against total human HER3/ERBB3 mAb (2F12) and human Phospho-ERBB3 (Y1262) were from Thermo Scientific Co. (Rockford, IL) and R&D systems, respectively. Beta-actin antibody (AC-15) and HSP70 antibody (W27) were from Santa Cruz biotechnology (Santa Cruz, CA) and Novus (Littleton, CO), respectively. HER2Mab was constructed as a recombinant human IgG1 based on variable domains of the anti-HER2 antibody rhuMAB 2C4 (pertuzumab) [7] and expressed transiently in HEK293 cells and purified based on protocols described previously [10]. MCF7 and MCF7-HER2 cells were grown in RPMI1640 supplemented with 10% FBS, 2 mmol/L glutamine, and antibiotics (50 units/mL penicillin and 50 μg/mL of streptomycin) in a humidified atmosphere of 5% CO_2_ at 37°C.

### Antibody and drug treatment, cell lysis, and Western blotting

MCF7 and MCF7-HER2 cells were seeded in 12-well plates and cultured for 12 h in RPMI1640 supplemented with 1% FBS (low serum contained media) before treatment. Cells were then treated with antibodies, such as HER2Mab in low serum containing media as indicated and stimulated with or without 100 ng/mL of NRG1 for 10 min or as indicated in the Figure legends. Medium was aspirated and cells were lysed with 100 μL/well of radioimmunoprecipitation assay (RIPA) buffer (Boston BioProducts, MA) and both phosphatase inhibitor cocktail II and III (Sigma-Aldrich, St Louis, MO) and protease inhibitor cocktail V (Calbiochem, San Diego, CA) were added right before preparation of cell lysates according to manufacturer's instruction. Protein concentration of cell lysate was measured with the BCA (bicinchoninic acid) protein assay reagent (Thermo Scientific). Lysates were mixed with sample loading buffer and boiled for 5 min before separation on polyacrylamide gel electrophoresis (PAGE) containing sodium dodecyl sulfate (SDS) separation system (Bio-Rad, CA). Proteins were transferred to a nitrocellulose membrane, and blocked with 5% bovine serum albumin (BSA) or 5% nonfat dry milk in TBST (Tris-buffered saline, containing 20 mmol/L Tris-HCl, pH 7.6, 137 mmol/L NaCl, and 0.5% Tween 20) overnight at 4°C. Blots were incubated with primary antibodies according to manufacturer's instructions and washed with TBST for three times and incubated with anti-mouse, anti-rat (Santa Cruz Biotechnolgoy), or anti-rabbit (Epitomics) secondary antibodies coupled with horse radish peroxidase (HRP) for 30 min. After washing three times with TBST, target proteins were detected with the Enhanced Chemiluminescence kit (Thermo Scientific). Films were scanned and bands were quantified using Image J software (NIH).

### NanoPro Immunoassay

A NanoPro 1000 instrument (Protein Simple, Santa Clara, CA) was used for the NanoPro Immunoassay (NIA). Cells were grown in 12-well plate until ∼80% confluency, serum starved for overnight and pretreated with the antibody (10 μg/mL) for 2 h and followed by NRG1 stimulation (100 ng/mL) for 10 min. Cells were lysed and protein concentration was measured by the BCA assay. Cell lysates were diluted to proper concentrations with sample diluent, mixed with Premix G2 4-7 or 5-8 (Protein Simple), including pI standard (#1 or #3, Protein Simple), and loaded into 384-well microplate. Samples were analyzed based on manufacturer's protocol. As an internal standard, HSP70 was detected with anti-HSP70 antibody (Novus).

### In situ PLA

MCF7 and MCF7-HER2 cells were grown on 8-well chamber slides until 70–80% confluency, and cells were serum starved in RPMI1640 containing 1% FBS overnight. Starved cells were pretreated with 10 μg/mL of HER2Mab and followed by treatment of NRG1 for 10 min. Cells were then washed with PBS, fixed in 4% paraformaldehyde in PBS for 30 min, permeabilized in 0.1% Triton X-100 for 20 min, and blocked with Duolink II blocking solution (Olink Biosiences, Uppsala, Sweden). After blocking, cells were incubated with combination of primary antibodies (anti-EGFR/HER3, anti-HER2/HER3, and anti-EGFR/HER2) in a preheated humidity chamber for 1 h at 37°C. Cells were then incubated with the PLA probes diluted 1:5 in antibody diluent (Olink) in a humidified chamber for 1 h at 37°C. Subsequent hybridization, ligation, amplification, and detection were performed using manufacturer's instruction (Olink). Fluorescence images were acquired using a Zeiss Axiovert microscope (Carl Zeiss Microscopy, Thornwood, NY).

### Cell proliferation assay

The xCELLigence RTCA system (Roche, Mannheim, Germany) was used to monitor cell index. Briefly, 5000 cells were seeded into a 96-well E-plate (Roche) in RPMI media with 1% FBS for 3 h to allow cells to attach on the bottom of each well. Treatment started by adding 10 μg/mL of HER2Mab and 100 ng/mL of NRG1 and cell growth was monitored for 5–7 days continuously.

### Cell migration assay

After MCF7 and MCF7-HER2 cells were trypsinized, cells (1 × 10^6^) were resuspended in RPMI containing 0.1% BSA, 10 μg/mL of HER2Mab was added to the top of Transwell (Corning, NY) migration chamber (6 well, 8 μm pore) and allowed to migrate for 18 h in the presence of RPMI containing 10% FBS. Residual cells were removed from top of the membrane with cotton ball and the cells on the underside of membrane were stained with crystal violet for 5 min. The cells that stained were photographed and counted from six random fields (×10) using a bright field microscope.

### Statistical analysis

All experiments were repeated at least three times and statistical significance was estimated using Student's *t*-test. *P*-value less than 0.05 between two treatments is considered significant.

## Results

### HER2Mab-stimulated MCF7 breast cancer cell proliferation independent of the HER3 ligand neuregulin 1 (NRG1)

**Figure 1:**
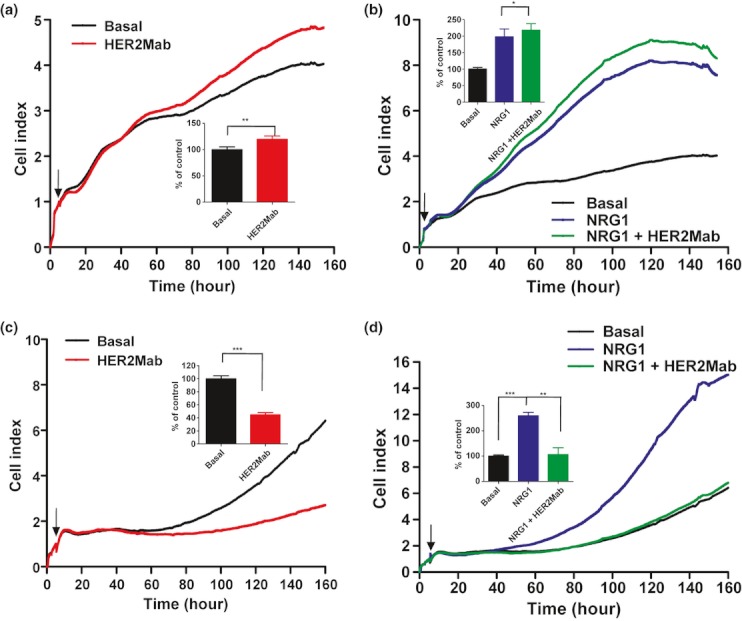
HER2Mab-induced cell proliferation of MCF7 cells in the absence of NRG1. MCF7 and MCF7-HER2 cells were trypsinized and seeded at 5000 cells/well in low serum (1%) medium in an E-plate (Roche). Cells were allowed for attachment to the plate for 3 h. HER2Mab (10 μg/mL) and/or NRG1 (100 ng/mL) were added according to the study design. Arrows indicated starting point of treatment. Cell proliferation was monitored label-free for 144 h at 37°C with an xCELLigence instrument (Roche). Both cell growth index curves and cell growth as percent of control at 144 h were presented. Each treatment contains eight replications and standard errors are shown on the bar graphs. Student's *t*-test was applied for pairwise statistical comparisons, *P < 0.05; **P < 0.01; ***P < 0.001. (a) MCF7 cells treated with HER2Mab; (b) MCF7 cells treated with NRG-1 and HER2Mab/NRG1; (c) MCF7-HER2 cells treated with HER2Mab; and (d) MCF7-HER2 cells treated with NRG1 and HER2Mab/NRG1.

MCF7 is known to express low levels of EGFR, HER2, and HER3 [8], but all three receptors were detectable on the cells by immune florescence staining with ERBB specific antibodies (Fig. S1a). Flow cytometry method also showed low, but detectable HER2 expression in MCF7 cells, and as expected MCF7-HER2 cells exhibited high level of HER2 expression (Fig. S1b). Cell proliferation of MCF7 and MCF7-HER2 under different treatment conditions was monitored continuously for 144 h using the xCELLigence instrument (Roche). Cell index, which is a measurement of cell proliferation, at the end of the study is plotted for statistical analysis. HER2Mab, which was constructed as a human IgG1 based on variable domains of the anti-HER2 antibody rhuMAB 2C4 (pertuzumab) and purified using protein A affinity resin (Fig. S2) [7], showed a subtle, but significant increase in MCF7 cell proliferation over the basal level in the absence of NRG1 (Fig. 1a). This unexpected increase in cell proliferation by HER2Mab was not visible until 60 h into the treatment (Fig. 1a). Treatment with the HER3 ligand neuregulin1 (NRG1) doubled MCF7 cell proliferation when compared with the basal control (Fig. 1a). More strikingly, the combined NRG1 and HER2Mab treatment showed additive increase in MCF7 cell proliferation (Fig. 1b).

To study the effect of HER2Mab and NRG1 on proliferation of high HER2 expressing cells, similar experiments were performed using MCF7-HER2 cells. In contrast to the MCF7 cells, HER2Mab significantly inhibited proliferation of MCF7-HER2 cells in the absence of NRG1 (Fig. 1c). As expected, NRG1 significantly increased MCF7-HER2 cell proliferation, which reached more than two-fold above the basal control, and HER2Mab neutralized the NRG1-induced cell proliferation to the basal level (Fig. 1d).

### HER2Mab-induced cell migration of MCF7 cells in the presence and absence of NRG1

**Figure 2:**
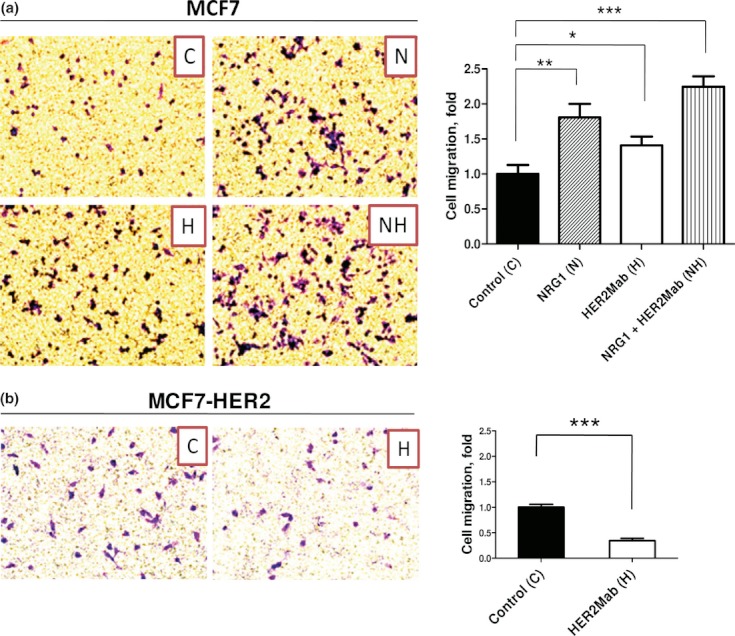
HER2Mab-induced cell migration of MCF7 cells in the absence of NRG1. Trypsinized MCF7 (a) or MCF7-HER2 (b) cells (1 × 10^6^) were seeded into Transwell plate (Costar, Corning, NY), and treated with PBS (c), NRG1 (100 ng/mL) (N), 10 μg/mL of HER2Mab (H), or combined NRG1 and HER2Mab treatment (NH). After 18 h incubation at 37°C, migrated cells were stained with 0.5% crystal violet for 5 min and visualized under a Carl Zeiss fluorescence microscope. Each treatment contains eight replications and standard errors are shown on the bar graph. Student's *t*-test was applied for pairwise statistical comparisons, *P < 0.05; **P < 0.01; ***P < 0.001. C, basal migration; N, cells treated with NRG1; H, cells treated with HER2Mab; NH, cells under the combined treatment of NRG1 and HER2Mab.

To study the effect of HER2Mab on migration of the low HER2 expressing MCF7 cells in the presence and absence of NRG1, cells were treated with HER2Mab in a transwell cell migration assay. Number of cells migrated crossing the membrane from the upper chamber of transwell is shown in the left-side images and the right-side bar graph shows the statistical comparison of cell migration among the treatments (Fig. 2a). HER3 ligand NRG1 increased MCF7 cell migration about two-fold above the basal control, and HER2Mab also enhanced cell migration to 1.5-fold over the basal control in MCF7 cells (Fig. 2a) in the absence of NRG1. The combined HER2Mab and NRG1 treatment showed additive effect on cell migration of MCF7 cells (Fig. 2a). In contrast, HER2Mab effectively blocked MCF7-HER2 cell migration (Fig. 2b).

### HER2Mab-stimulated ligand-independent EGFR/HER3 dimerization in MCF7 cells

**Figure 3:**
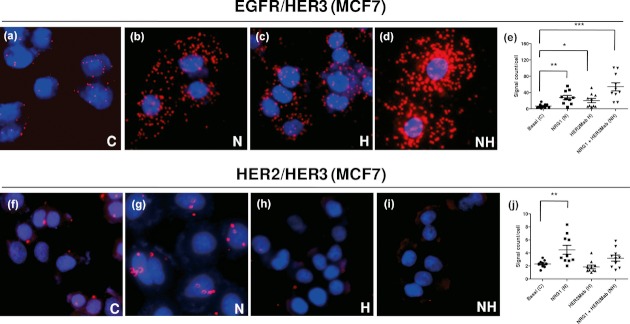
HER2Mab-induced ligand-independent EGFR/HER3 dimerization in MCF7 cells. HER3/EGFR or HER3/HER2 dimerization was visualized by the proximity ligation assay (PLA) protocol [11]. After serum starved with low serum media (1%) for 24 h, MCF7 cells were treated with PBS (C), NRG1 (N), HER2Mab (H), or combined HER2Mab/NRG1 (NH). After performing the PLA procedure, images were taken with a Carl Zeiss Fluorescence microscope, red spots which represent individual dimers from 10 randomly selected images were counted and plotted. Student's *t*-test was applied for pairwise statistical comparisons between treatments, *P < 0.05; **P < 0.01; ***P < 0.001. (a) A representative image of basal EGFR/HER3 dimerization in MCF7 cells. (b) EGFR/HER3 dimerization in MCF7 cells treated with NRG1. (c) EGFR/HER3 dimerization in MCF7 cells treated with HER2Mab. (d) EGFR/HER3 dimerization in MCF7 cells treated with HER2Mab and NRG1. (e) Statistical analysis of treatments (a–d). (f) Basal HER2/HER3 interaction in MCF7 cells. (g) HER2/HER3 interaction in MCF7 cells treated with NRG1. (h) HER2/HER3 interaction in MCF7 cells treated with HER2Mab. (i) HER2/HER3 interaction in MCF7 cells treated with HER2Mab and NRG1. (j) Statistical analysis of treatments (f–i).

To understand the signaling mechanism of HER2Mab-induced MCF7 cell proliferation and migration, HER3/HER2, HER3/EGFR, HER2/EGFR receptor dimerization was measured using an immunoblotting/molecular imaging method known as the PLA [11]. Basal EGFR/HER3 dimers were minimally detected in MCF7 cells in the absence of NRG1 (Fig. 3a). As expected, the HER3 ligand NRG1 significantly induced EGFR/HER3 dimerization in MCF7 cells (Fig. 3b). More importantly, HER2Mab also significantly induced EGFR/HER3 dimerization in the absence of NRG1 in MCF7 cells (Fig. 3c), and the combined NRG1 and HER2Mab treatment induced significantly higher EGFR/HER3 dimerization than NRG1 or HER2Mab alone in MCF7 cells (Fig. 3d). Figure 3e is the statistical analysis of images shown in Figure 3a–d. NRG1 treatment also stimulated HER2/HER3 dimerization over the basal control in MCF7 cells, even though density of HER2/HER3 dimers was much lower than that for EGFR/HER3 dimers (Fig. 3f and g). However, HER2Mab completely blocked HER2/HER3 dimerization in the presence and absence of NRG1 (Fig. 3h and i). These results suggest that blockage of HER2/HER3 dimerization by HER2Mab triggered HER3/EGFR dimerization for compensatory signaling. Figure 3j is the statistical analysis of images shown in Figure 3f–i.

**Figure 4:**
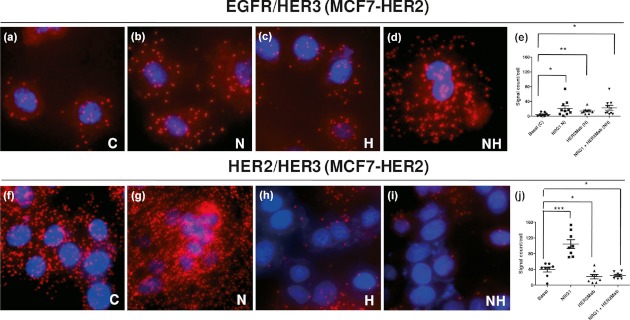
HER2Mab effectively blocked HER2/HER3 dimerization in MCF7-HER2 cells. HER3/EGFR or HER3/HER2 dimerization was visualized by the proximity ligation assay (PLA) protocol [11]. After serum starved with low serum media (1%) for 24 h, MCF7-HER2 cells were treated with NRG1 (N), HER2Mab (H), or combined HER2Mab/NRG1 (NH). After performing the PLA procedure, images were taken with a Carl Zeiss Fluorescence microscope, red spots which represent individual dimers from 10 randomly selected images were counted and plotted. Student's *t*-test was applied for pairwise statistical comparisons between treatments, *P < 0.05; **P < 0.01; ***P < 0.001. (a) A representative image of basal EGFR/HER3 dimerization in MCF7-HER2 cells. (b) EGFR/HER3 dimerization in MCF7-HER2 cells treated with NRG-1. (c) EGFR/HER3 dimerization in MCF7-HER2 cells treated with HER2Mab. (d) EGFR/HER3 dimerization in MCF7-HER2 cells treated with HER2Mab and NRG1. (e) Statistical analysis of treatments (a–d). (f) Basal HER2/HER3 interaction in MCF7-HER2 cells. (g) HER2/HER3 interaction in MCF7-HER2 cells treated NRG1. (h) HER2/HER3 interaction in MCF7-HER2 cells treated with HER2Mab. (i) HER2/HER3 interaction in MCF7-HER2 cells treated with HER2Mab and NRG1. (j) Statistical analysis of treatments (f–i).

In the HER2 overexpressing MCF7-HER2 cells, basal EGFR/HER3 dimers were readily detected in the absence of NRG1 (Fig. 4a), and NRG1 significantly stimulated EGFR/HER3 dimerization (Fig. 4b). Similar to MCF7 cells, MCF7-HER2 cells also increased EGFR/HER3 dimerization when treated with HER2Mab (Fig. 4c), and the combination of NRG1 and HER2Mab treatment induced even higher level of EGFR/HER3 dimerization than NRG1 or HER2Mab alone in MCF7-HER2 cells (Fig. 4d). Figure 4e is the statistical analysis of images shown in Figure 4a–d. Not surprisingly, HER2/HER3 dimers are abundant in the absence of NRG1 in the high HER2 expressing MCF7-HER2 cells (Fig. 4f). HER3 ligand NRG1 significantly enhanced HER2/HER3 dimerization (Fig. 4g). As expected, HER2Mab effectively blocked ligand-independent and ligand-induced HER2/HER3 interactions in MCF7-HER2 cells (Fig. 4h and i). Figure 4j is the statistical analysis of images shown in Figure 4f–i. The opposite effects of HER2Mab on EGFR/HER3 and HER2/HER3 dimerization indicated that HER3 plays a central role in balancing the ERBB family protein signaling circuit.

It is noted that EGFR/HER2 dimerization was not detectable in either cell lines in the absence or presence of NRG1, and HER2Mab treatment had no effect on EGFR/HER2 dimerization (Fig. S3). These results indicate that HER3 is a preferred partner for HER2 and EGFR in both MCF7 and MCF7-HER2 cells.

### HER2Mab-induced ligand-independent HER3 and AKT phosphorylation in MCF7 cells

**Figure 5:**
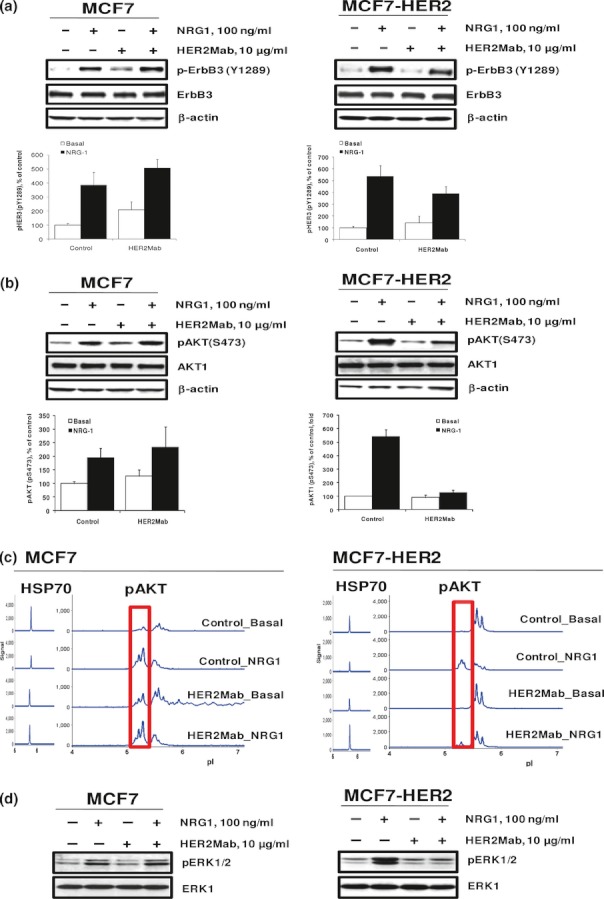
HER2Mab-induced ligand-independent HER3 phosphorylation and AKT phosphorylation in MCF7 and MCF7-HER2 cells. (a) p-HER3(Y1289) in MCF7 and MCF7-HER2 cells in response to NRG1 and HER2Mab treatment. Western gel images showed were quantitated using the ImageJ software after multiple images were taken from separate experiments (*n* = 3). (b) pAKT(S473) in MCF7 and MCF7-HER2 cells in response to NRG1 and HER2Mab treatment as determined by Western blot. (c) pAKT(S473) in MCF7 and MCF7-HER2 cells in response to NRG1 and HER2Mab treatment as determined using the nanofluidic immunoassay. Peaks in the boxed area indicate pAKT(S473). (d) pERK1/2 in MCF7 and MCF7-HER2 cells in response to NRG1 and HER2Mab treatment as determined by Western blot.

To investigate whether the HER2Mab-induced proliferation and migration of MCF7 cells is in part mediated by ligand-independent HER3 activation and downstream signaling, phosphorylation of HER3, AKT, and ERK was determined by Western blotting after treatment with HER2Mab in the absence or presence of NRG1. Phosphorylation of HER3 (pHER3-Y1289) was highly induced by NRG1 in both MCF7 and MCF7-HER2 cells (Fig. 5a). HER2Mab slightly stimulated pHER3 (Y1289) of MCF7 cells in the absence of NRG1, and the combination of NRG1 and HER2Mab treatment showed greater enhancement on HER3 activation as indicated by pHER3 (Y1289) (Fig. 5a). In contrast, HER2Mab did not increase pHER3(Y1289) in MCF7-HER2 cells (Fig. 5a). HER2Mab showed no effect on pHER3(Y1289) in the presence of NRG1 (Fig. 5a), which suggests that HER2Mab forced HER3 to dimerize with EGFR and the HER3 phosphorylation is a result of HER3/EGFR interaction.

It is well documented that HER3 activation induces the downstream AKT signaling pathway. In MCF7 cells, NRG1 significantly induced pAKT (S473) and HER2Mab also slightly stimulated AKT phosphorylation in the absence of NRG1, and the combination of NRG1 and HER2Mab treatment further enhanced AKT activation (Fig. 5b). As expected, NRG1-induced pAKT(S473) in both low HER2 and HER2 overexpressing MCF7 cancer cell lines, and the response in MCF7-HER2 was more profound (Fig. 5b). To confirm the Western blot results, we further interrogated the effect of HER2Mab on AKT phosphorylation using the more sensitive nanofluidic immunoassay which detects the overall phosphorylation state of AKT [12]. NRG1, HER2Mab, and combined NRG1/HER2Mab-induced AKT phosphorylation of MCF7 cells as indicated by the left-shift of multiple peaks in the boxed areas (Fig. 5c). In contrast, HER2Mab effectively blocked AKT phosphorylation in the presence or absence of NRG1 in MCF7-HER2 cells which overexpress HER2, but HER2Mab increased slightly pAKT in the low HER2 MCF7 cells (Fig. 5b and c). The opposite effects of HER2Mab on AKT phosphorylation in MCF7 and MCF7-HER2 cells indicate that the impact of HER2Mab on HER2 signaling depends on HER2 expression status. Consistent with the EGFR/HER3 and HER2/HER3 dimerization results, HER2Mab did not inhibit pAKT (S473) and pERK1/2 in the presence or absence of NRG1 in MCF7 cells (Fig. 5d), but showed clear inhibition of pAKT and pERK1/2 in MCF7-HER2 cells (Fig. 5d).

## Discussion

Molecular cancer therapies targeting EGFR and HER2 have been successfully applied in the clinic for treatment of different cancers [13]. However, resistance to the therapies is wide spread and complex compensatory ERBB signaling directly contributes to the resistance. Unlike EGFR, HER2, and HER4 which possess active tyrosine kinase domains, HER3 lacks intrinsic kinase activity [14] and HER3 homodimer has not been reported [15]. HER3 activation was reported in many other cancer types, such as melanoma, breast, pancreatic, prostate, ovarian, and gastric cancers [16–21], but our understanding on HER3's role in cancer biology is limited. Activation of HER3 is believed to rely on ligand binding and dimerization with other ERBB family members. Among all the homodimer and heterodimer pairs, HER3/HER2 dimerization is the most potent partner for activation of the PI3K/AKT signaling cascade through direct HER3 binding to the p85 subunit of PI3K [22, 23]. Growing evidence suggests that HER3 plays a pivotal role in regulation of the ERBB signaling cascade [24–29], and compensatory HER3 phosphorylation leads to resistance to current ERBB molecular targeting agents in vitro and in vivo [30–32]. The results in this study showed HER3 activation upon HER2Mab treatment in both MCF7 (low HER2) and MCF7-HER2 (high HER2) cancer cells independent of the HER3 ligand NRG1, and HER3 activation is a result of HER3/EGFR dimerization. Similar increase in HER3/EGFR dimerization by HER2Mab was also observed in T47D (a low HER2 expressing cancer cell line) and SKBR3 (a high HER2 expressing cancer cell line) (Fig. S4).

Dimerization of HER family proteins is a prerequisite for activation of the ERBB signaling network. Among dimers of the ERBB family members, HER2/HER3 is the preferred dimerization partner and this is especially true in high HER2 expressing cells [33]. As MCF7 cells express low levels of HER2, HER2/HER3 dimerization may not be the dominant interaction. Not surprisingly, HER2/HER3 dimers are abundant in the HER2 overexpressing MCF7-HER2 cells in the absence of NRG1 and this is consistent with previous report that ligand-independent HER2/HER3 dimerization occurs in high HER2 expressing cancer cells [4].

MCF7 cells are known not to express the two HER3 ligands NRG1 and NRG2 [34] and the RT-PCR experiments conducted in this study confirmed the previous findings (Fig. S5). The lack of endogenous expression of HER3 ligands made MCF7 a suitable cell line to study ligand-dependent and ligand-independent HER3 signaling. HER2Mab is constructed as an IgG1 antibody based on variable domains of the anti-HER2 antibody rhuMAB 2C4 (pertuzumab) [7]. rhuMAB 2C4 is known to inhibit proliferation of HER2 overexpressing cancer cells via blockage of HER2 dimerization with other ERBB proteins [35–37]. The results of blocking HER2/HER3 dimerization by HER2Mab in MCF7-HER2 cells suggest that the recombinant HER2Mab used in this study is functionally similar to rhuMAB 2C4. Studies have shown that rhuMAB 2C4 was not capable of inhibiting proliferation of low HER2 cancer cells including MCF7 cells [8, 9, 38, 39]. However, our results demonstrated that HER2Mab not only did not inhibit MCF7 cell proliferation, but it promoted proliferation of the low HER2 expressing MCF7 breast cancer cells in the absence of HER3 ligand NRG1. One possible explanation is that the label-free xCELLigence assay provided a more sensitive cell proliferation detection method than the traditional end point assays, and the end point cell proliferation assays at fixed time points may have missed the subtle changes in cell proliferation. Careful examination of a previous report indeed showed an about 10% increase in MCF7 cell proliferation by pertuzumab [9].

Demonstration of increased phosphorylation and activation of HER3 and AKT signaling by HER2Mab in MCF7 cells in the absence of NRG1 suggests that the HER2Mab-induced, ligand-independent HER3 dimerization with EGFR (HER3/EGFR) is functional in triggering downstream signaling which results in enhanced MCF7 cell proliferation and migration. Similarly, HER2/HER3 dimerization was detected in the high HER2 context of MCF7-HER2 cells in the absence of NRG1. Ligand-independent dimerization among the ERBB proteins have been recently reported [4, 40], but this is the first study on the detection of EGFR/HER3, and HER2/HER3 dimerization in the absence of NRG1 in both low HER2 (MCF7) and high HER2 (MCF7-HER2) cell lines. These results suggest that ligand-independent dimerization among the ERBB proteins exists more widely than previously thought.

The effect of HER2Mab on MCF7-HER2 cell proliferation is in agreement with previous reports that rhuMab2C4 or pertuzumab potently inhibits proliferation of HER2 overexpressing breast cancer cell lines, such as BT474 and SKBR3 [39, 41]. The different responses of MCF7-HER2 and MCF7 cells to HER2Mab treatment might help us understand why pertuzumab showed efficacy in HER2-positive but not HER2-negative metastatic breast cancer patients [42, 43]. It has been reported that the HER2 targeting trastuzumab treatment can alter the breast tumor from high HER2 to low HER2, which results in resistance to trastuzumab [44]. Similarly, our study suggests that clinical resistance to pertuzumab may arise dependent on HER2 expression status. Overall this study underlines the complexity of targeting ERBB signaling for cancer therapy and provides a mechanistic rationale for combination therapy to overcome drug resistance when targeting the ERBB signaling pathway for cancer treatment.

## Supporting Information

**Figure S1.** Expression of EGFR, HER2, and HER3 in MCF7 and MCF7-HER2 cells.

**Figure S2.** HER2Mab expression and purification.

**Figure S3.** Effect of HER2Mab on the basal level EGFR/HER2 dimerization in MCF7 or MCF7-HER2 cells.

**Figure S4.** Effect of HER2Mab on the basal level of EGFR/HER3 and HER2/HER3 dimerization in T47D and SKBR3 cells.

**Figure S5.** No endogenous neuregulin (NRG1) expression was detected in MCF7 and MCF7-HER2 cells.
