# Supplementary material for: ERBB3 (HER3) is a key sensor in the regulation of ERBB-mediated signaling in both low and high ERBB2 (HER2) expressing cancer cells
Source: Cancer Med. 2012 Jul 15;1(1):28–38. doi: 10.1002/cam4.10 (PMC3544427; doi:10.1002/cam4.10)
Supplement: Supplementary file 1 [file cam40001-0028-SD1.doc]

Supporting information

Choi B.K., et al

Fig. S1. Expression of EGFR, HER2 (ERBB2) and HER3 (ERBB3) in MCF7 and MCF7-HER2 cells.

**(**a) EGFR, HER2 and HER3are detected in MCF7 cells by immunofluorescent staining. To detect EGFR, HER2 and HER3 in MCF7 cells, cells were trypsinized, seeded into 8-well chamber slide and incubated overnight at 37°C. Cells were then fixed with 4% paraformaldehyde and permeabilized with 0.1% of Triton X-100, blocked with 5% BSA and incubated with anti-EGFR (Epitomics, Cat # 1114-1), anti-HER2 (Epitomics, Cat #2064-1) and anti-HER3 antibodies (Thermo Scientific, Cat # MS-201-P1A). Receptors were detected using anti-mouse-Alexa Fluro® 518 and anti-rabbit-Alexa Fluor® 488 antibodies. Fluorescence images were acquired using a Zeiss Axiovert microscope (Carl Zeiss Microscopy, Thornwood, NY). (b) HER2 expression in MCF7 and MCF7-HER2 as determined by flow cytometry. Cells under the gray area which shifted to the right indicated HER2 expression level. Cells were detached from the culture plate with 1 mM EDTA, collected and washed with PBS-T. After centrifugation at 1,800 rpm for 5 min, cells were blocked with 3% BSA in PBS for 20 min at room temperature followed by incubation in 100 µl PBS-T containing an anti-HER2 antibody (trastuzumab) for 1 h at room temperature. After washing twice with PBS-T, HER2 was detected using a R-Phycoerythrin-conjugated AffiniPure F(ab)2 fragment of goat anti-human IgG (Fcγ fragment specific). Flow cytometry was performed with a Guava easyCyte HT (Millipore).

Fig. S2. HER2Mab expression and purification.

HER2Mab was expressed transiently in HEK293 cells. For transient transfection, a pair of plasmids carrying light and heavy chain ORFs was transfected using FuGene 6 (Roche Diagnostics, IN) and Opti-MEM medium (Invitrogen, CA). Transfected cells were incubated at 37°C in a 5% CO2 incubator. Medium was harvested between day 7–10 post transfection. Antibodies were purified using protein A/G affinity chromatography according to the manufacturer’s recommendation (Pierce, IL). The purified antibodies were analyzed for their concentration (absorption at OD280 nm), purity and homogeneity by SDS-PAGE. The antibody was loaded onto SDS-PAGE, and run under reduced and non-reduced conditions. (a) under reducing condition, HER2Mab shows its heavy chain and light chain. (b) under non-reducing condition, HER2Mab is shown in its intact IgG1 form with a molecular weight of about 150 KD.

Fig. S3. Effect of HER2Mab on the basal level EGFR/HER2 dimerization in MCF7 or MCF7-HER2 cells.

EGFR/HER2 dimerization was measured by the proximity ligation assay (PLA) protocol [1] in MCF7 and MCF7-HER2 cells under various treatment conditions. Cells were plated on 8-well chamber slide and grown until 80% confluency. After serum starved with low serum media (1%) for 24 h, MCF7 or MCF7-HER2 cells were treated with PBS (C), 10 µg/ml of HER2Mab (H) for 2h, followed by 10 min of NRG1 (N) stimulation at 100 ng/ml for 10 min. Cells were washed with PBS, fixed with 4% paraformaldehyde and permeabilized with 0.1% Triton X-100. After blocking, cells were incubated with combinations of anti-EGFR and anti-HER2 antibodies in a pre-heated humidity chamber for 1 h at 37°C. Cells were incubated with PLA probe anti-rabbit MINUS and anti-mouse PLUS diluted 1:5 in antibody diluent (Olink Biosciences) in a humidified chamber for 1h at 37°C. Subsequent hybridization, ligation, amplification and detection were performed by manufacturer’s instruction (Olink Biosciences). Images were taken with a Carl Zeiss fluorescence microscope.

Fig. S4. Effect of HER2Mab on the basal level of EGFR/HER3 and HER2/HER3 dimerization in T47D and SKBR3 cells.

EGFR/HER3 and HER2/HER3 dimerization were visualized by the proximity ligation assay (PLA) protocol [1] in T47D and SKBR3 cells. Cells were plated on 8-well chamber slide and grown until 80% confluency. After serum starved with low serum media (1%) for 24 h, T47D (a) and SKBR3 (b) cells were treated with PBS (C), 10 µg/ml of HER2Mab (H) for 2h at 37°C. Cells were washed with PBS, fixed with 4% paraformaldehyde and permeabilized with 0.1% Triton X-100. After blocking, cells were incubated with combinations of anti-EGFR and anti-HER3, and anti-HER2 and anti-HER3 antibodies in a pre-heated humidity chamber for 1 h at 37°C. Cells were incubated with PLA probe anti-rabbit MINUS and anti-mouse PLUS diluted 1:5 in antibody diluent (Olink Biosciences) in a humidified chamber for 1h at 37°C. Subsequent hybridization, ligation, amplification and detection were performed by manufacturer’s instruction (Olink Biosciences). Images were taken with a Carl Zeiss fluorescence microscope.

Fig. S5. No endogenous neuregulin 1 (NRG1) expression was detected in MCF7 and MCF7-HER2 cells.

Expression of NRG1 mRNA by MCF7, MCF7-HER2, and MDA-MB-231 cells under various treatment conditions as determined by RT-PCR. MDA-MB-231 is known to express endogenous neuregulin 1 and was used as a positive control [2]. Lane assignments: **M**, 1kb ladder; **1**, MDA-MB-231; **2**, MCF7; **3**, MCF7 treated with HER2Mab; **4**, MCF7-HER2; and **5**, MCF7-HER2 treated with HER2Mab. Cells were plated in 6-well plates and grown to 70% confluency. Cells were synchronized for 24 h in RPMI1640 supplemented with 1% FBS (low serum containing media) and washed three times with phosphate buffered saline (PBS). HER2Mab treatment was for 2 h at 37°C. After cells were washed with PBS, total RNA was isolated using Trizol reagent (Invitrogen), 1 µg of RNA was used to synthesize first strand cDNA with superscript SK II (Invitrogen) and the following RT-PCR was performed in a 50 µl reaction volume: 95°C for 5 min then 95°C for 30 seconds, 55°C for 45 second, 72°C for 1 min, for 30 cycles, and extended at 72°C for 7 min. The primers to human NRG1 were as follows: NRG1, forward, 5′-gctagctagcatgtccgagcgcaaagaaggcaga-3′, and reverse 5′-gccgccggatccgccgccgccttcaggcagagaca ggggagtgga-3′. Beta-actin was used as an internal standard and its mRNA was amplified with primers: forward, 5′-ctcttccagccttccttcct-3’, and reverse 5′-caccttcaccgttccagttt-3’.

**References**

**[1] Soderberg O, Gullberg M, Jarvius M, Ridderstrale K, Leuchowius KJ, Jarvius J, Wester K, Hydbring P, Bahram F, Larsson LG, Landegren U. Nat Methods 2006;3(12):995-1000.**

**[2] Dunn M, Sinha P, Campbell R, Blackburn E, Levinson N, Rampaul R, Bates T, Humphreys S, Gullick WJ. J Pathol 2004;203(2):672-680.**
